# Warming and Elevated CO_2_ Interact to Drive Rapid Shifts in Marine Community Production

**DOI:** 10.1371/journal.pone.0145191

**Published:** 2015-12-29

**Authors:** Cascade J. B. Sorte, Matthew E. S. Bracken

**Affiliations:** Department of Ecology & Evolutionary Biology, 321 Steinhaus Hall, University of California Irvine, Irvine, California 92697–2525, United States of America; Universidad de la Republica, URUGUAY

## Abstract

Predicting the outcome of future climate change requires an understanding of how alterations in multiple environmental factors manifest in natural communities and affect ecosystem functioning. We conducted an *in situ*, fully factorial field manipulation of CO_2_ and temperature on a rocky shoreline in southeastern Alaska, USA. Warming strongly impacted functioning of tide pool systems within one month, with the rate of net community production (*NCP*) more than doubling in warmed pools under ambient CO_2_ levels relative to initial *NCP* values. However, in pools with added CO_2_, *NCP* was unaffected by warming. Productivity responses paralleled changes in the carbon-to-nitrogen ratio of a red alga, the most abundant primary producer species in the system, highlighting the direct link between physiology and ecosystem functioning. These observed changes in algal physiology and community productivity in response to our manipulations indicate the potential for natural systems to shift rapidly in response to changing climatic conditions and for multiple environmental factors to act antagonistically.

## Introduction

Recent climatic changes have been small relative to those expected in the future [1] yet have altered biological systems worldwide [2]. However, despite the increasing confidence with which climate modelers are making prognoses for future climate change [1], ecologists lack crucial biological data necessary to forecast future impacts on the earth’s species. In marine systems, few studies have explored the impacts of alterations in multiple environmental factors on natural communities, and even fewer have assessed the consequences of such changes for rates of ecosystem functioning; we address both of these issues in this study.

Species can be affected by climate change both directly (e.g., if mortality is elevated under increasingly stressful climatic conditions) and/or indirectly *via* changes in the abundance or *per capita* effects of interacting species. A recent meta-analysis showed that marine species’ responses to acidification differed when they were measured in studies with single versus multiple species [3]. Furthermore, interacting species often respond differently when subjected to changes in multiple climate factors at the same time [4]. Temperature and pH can affect species interactively, either amplifying or reducing both positive and negative responses. The few previous studies of these multiple stressors in marine systems have, more often than not, demonstrated an interaction between increased temperature and increased CO_2_ / decreased pH [4], and interactive effects have been highly variable in both magnitude and direction [3]. Thus, in order to predict future impacts of climate change, we need to consider responses to multiple, interacting environmental factors, and how these responses manifest *in situ*, in the context of a natural community.

Field manipulations of multiple environmental factors have the potential to provide some of the best predictions of how climate change will impact coastal systems. Empirical, community-level field data are notably lacking in marine systems as compared to the more numerous results from terrestrial warming and FACE (Free-Air CO_2_ Enrichment) experiments [5]. The majority of marine climate-change studies have (i) used observational (rather than experimental) methods, (ii) examined a single environmental factor, and (iii) focused on a single species [6]. A more recent review of marine climate-change studies conducted between 2000 and 2009 showed that these same gaps remain [7]. Specifically, only 35% of studies reviewed included multiple climate variables, 19% quantified impacts at the community level, and 11% included some type of field assessment (transplant or space-for-time techniques were most common; none were replicated manipulative experiments of environmental factors). The few temperature manipulations in coastal systems have used passive warmers [8] or heated tiles [9]. CO_2_ has been manipulated in a few subtidal systems (e.g., [10]) and, as far as we know, only two intertidal communities [11,12]. Thus, whereas climate change has been identified as one of the primary drivers of global biodiversity loss [13], impacts on marine ecosystems remain poorly understood.

**Fig 1:**
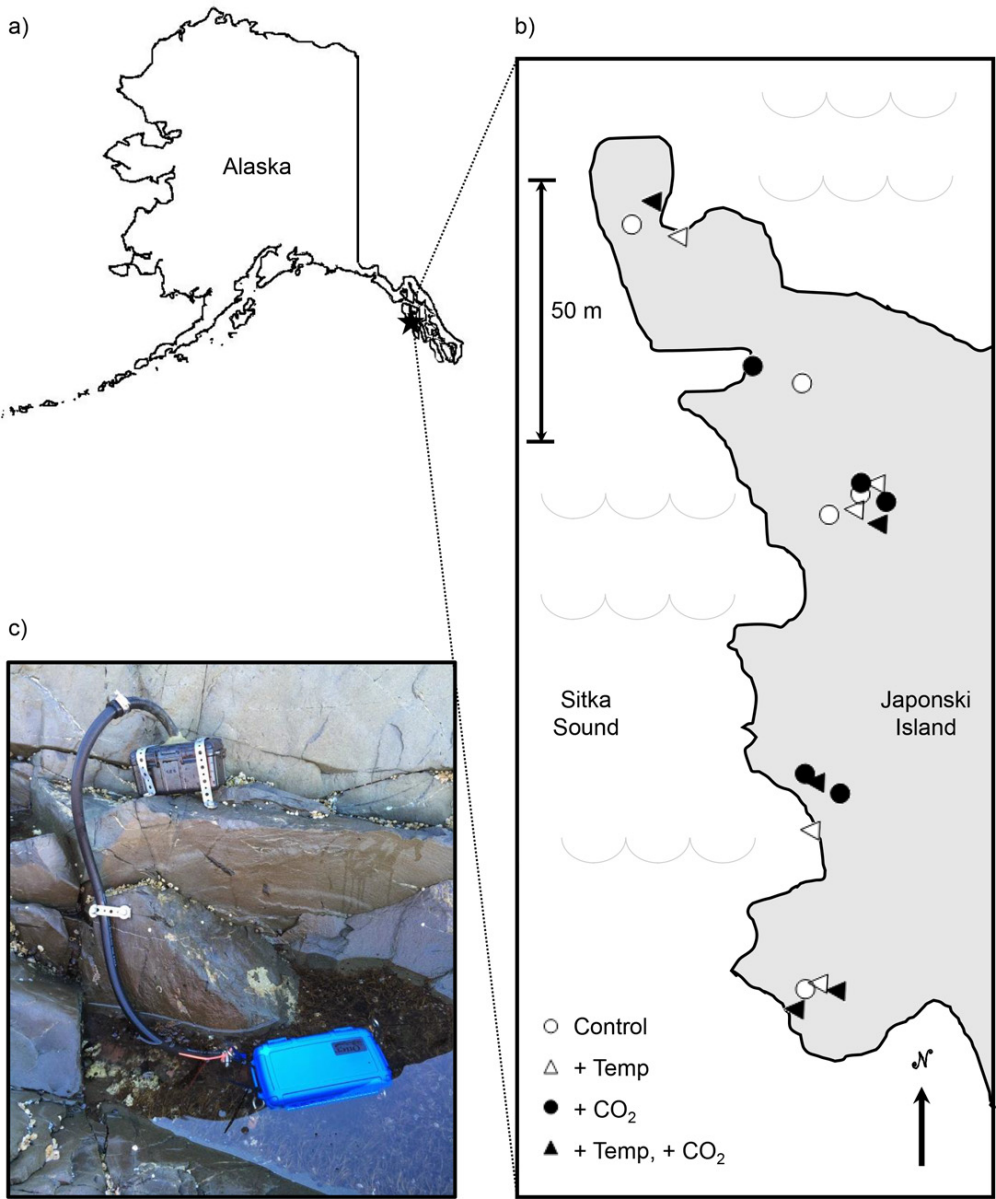
Field site and experimental design. (a) Location of field site in Sitka, Alaska, USA (map redrawn from the USGS TNM 2.0 viewer [public domain]). (b) Tide pools (n = 20) were randomly assigned to treatment: control (open circle), +Temp (open triangle), +CO_2_ (closed circle), and both (closed triangle). (c) Representative tide pool. The submerged OtterBox® contained a warmer, and the OtterBox® outside the pool contained a yeast solution that generated CO_2_ which bubbled into the pool.

Here, we describe a fully factorial field experiment in which we manipulated environmental conditions by increasing CO_2_ and temperature in natural tide pool habitats in southeastern Alaska (Fig 1). We measured biological metrics from the organismal (i.e., seaweed nutrient ratios) to the ecosystem (i.e., net community production) level both before and after warming and CO_2_ addition to test the hypothesis that these climate factors would have individual and combined effects on the composition, diversity, and productivity of a natural marine community. We predicted that adding CO_2_ would enhance net community production (due to potential carbon limitation in tide pools) and that warming effects would be either positive (due to increased metabolic rates) or negative (due to increased thermal stress).

## Materials and Methods

Our field site was John Brown’s Beach on Japonski Island, Sitka, Alaska, USA (57.06°N, 135.37°W; Fig 1A). Our research and collections at the site were conducted with the permission of the Alaska Department of Fish and Game (Permit CF-14-098). The site is characterized by a maximum tidal amplitude of 6.48 m. We identified 20 pools in the mid-to-high intertidal zone (Fig 1B). For each pool, we determined tide height (2.51 ± 0.05 m above mean lower low water; all values given as mean ± 1 SE), volume (11.31 ± 1.7 L), perimeter (2.43 ± 0.21 m), and maximum depth (12.3 ± 1.0 cm). Pools were randomly assigned to one of the four treatments (n = 5 per treatment): control (un-manipulated), +CO_2_ (CO_2_ added), +Temp (warmed), and both (CO_2_ added and warmed). Assignments were re-randomized until the following pool attributes did not differ significantly between treatments: volume, surface area, and initial values for sessile species cover, sessile species richness, and mobile species richness (for all, ANOVA p > 0.1).

Tide pool temperatures and CO_2_ levels were manipulated between 17 July and 01 August 2014. To simulate a climate-related temperature increase during the most stressful part of the tidal cycle–the midday low tide–when pools are naturally warmest, tide pool temperature was manipulated using rechargeable hand warmers (EnerHandz^®^) packaged in waterproof OtterBoxes® and attached to the bottom of the pools. Unwarmed pools contained empty OtterBoxes® to control for potential effects of shading or disturbance. Warmers were replaced daily, and pool temperatures were recorded every 10 min by TidbiT^®^ data loggers (Onset^®^, Bourne, Massachusetts, USA). CO_2_ was delivered to each elevated CO_2_ pool *via* tubing from a yeast reactor [14]: a watertight plastic box (Drybox 2500, OtterBox, Fort Collins, Colorado, USA) containing 500 mL of water, 75 g of sugar, 2 g of yeast, and 2 g of NaHCO_3_ to buffer the internal pH of the reactor (Fig 1C). CO_2_ is the most abundant gas produced during fermentation of baker’s yeast and is produced by this mixture at a rate of approx. 140 ml CO_2_ per min [15]. The reactor solutions were replaced every 3–4 days, and tide pool pH was measured daily (while gently stirring the pool) with a handheld pH meter (pH10A, YSI, Yellow Springs, Ohio, USA). Measurements of pH are presented on the total hydrogen ion concentration scale after cross-calibration with buffers prepared according to Dickson [16].

**Fig 2:**
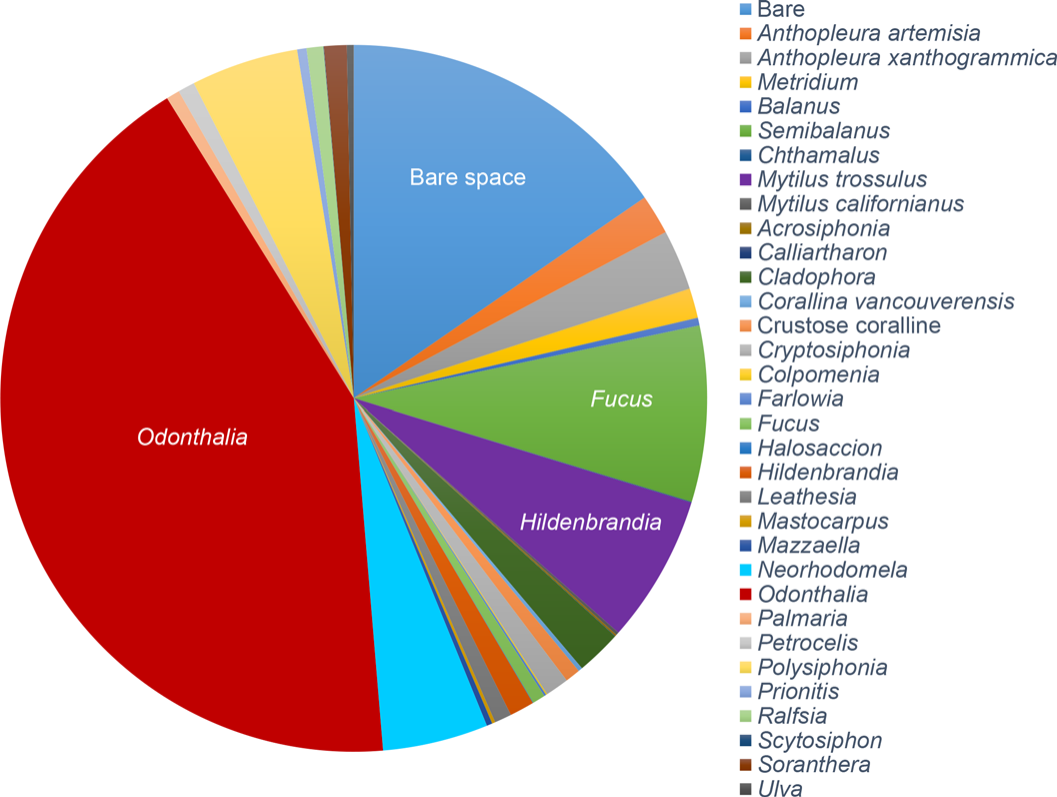
Relative cover of sessile species in experimental tide pools. The red alga *Odonthalia floccosa* was the most abundant species, covering 48.27% (± 0.06 SE) of available space. 17.49% (± 0.04 SE) of space was bare. Values are mean percent cover across the n = 20 pools.

Both before and after the experimental manipulations, we conducted community surveys, measured rates of productivity, and collected samples of *Odonthalia floccosa* (Esper) Falkenberg (hereafter, *Odonthalia*), the most common seaweed species (covering 48.27 ± 0.06% of available space, Fig 2) and the only seaweed species present in all pools. Algal samples (one thallus per pool) were washed in deionized water, blotted dry, and stored at -25°C. For analyses of internal C:N, samples were dried to constant mass (60°C for 72 h), ground to a fine powder (Retsch Mixer Mill MM 400, Verder Scientific, Newtown, Pennsylvania, USA) and analyzed on a Flash 2000 Elemental Analyzer (Thermo Fisher, Cambridge, UK).

On 10–12 July 2014 and 02 August 2014, we quantified percent cover of sessile species (macroalgae and sessile invertebrates) and counted mobile invertebrates in emptied pools by laying a flexible mesh quadrat across the bottom surface area. Water was retained in an adjacent bucket and replaced within approx. 10 min to limit stress to tide pool organisms. We measured net community production on 15 and 31 July 2014 by quantifying the change in O_2_ concentrations (using a Professional Plus Multiparameter Meter, YSI, Yellow Springs, Ohio, USA) in the pools in the light according to published methods [17]. To ensure that initial oxygen concentrations were low, we took our first sample after pools were covered with dark plastic for ~30 min. We took a second sample after ~30 min of exposure to daylight at saturating irradiance levels (703 ± 115 [mean ± SE] μmol photons m^-2^ s^-1^). Pools were gently stirred with the multimeter probe prior to O_2_ measurements to prevent stratification. Net community production rates (*NCP*) were calculated as follows:

$$NCP\;=\;|\;\Delta {\left[O_{2}\right]}_{light}\;/\;\Delta t_{light}\;|$$ (1)

where Δ[*O*_2_] is the change in the O_2_ concentration (mg O_2_ L^-1^) and Δ*t* is the change in time.

To compare temperatures in experimentally warmed tide pools with those in un-warmed, ambient pools, we determined the 90^th^ percentile of temperatures recorded by our TidbiT^®^ data loggers each day in each tide pool. We calculated the difference between the average temperatures of ambient and warmed pools on each day of our experiment and used linear regression (PROC GLM in SAS v. 9.4, Cary, North Carolina, USA) to examine this difference as a function of the 90^th^ percentile of air temperatures for each day (recorded by the weather station at the Sitka Airport, < 1 km from our study site). To compare pH in our ambient and CO_2_ addition pools, we used a two-sample *t*-test of the mean daily pH values recorded in every pool 1.4 ± 0.3 h before the daytime low tide. Note that because CO_2_ was bubbled continuously into pools, differences between ambient and CO_2_ addition pools were likely to increase over the course of a low tide, with maximum differences immediately prior to re-submersion by the incoming tide.

We used general linear models (ANOVA, repeated measures ANOVA) to evaluate effects of our experimental treatments on *NCP*, C:N, and *Odonthalia* cover. Other analyses included *t*-tests to evaluate temperature and pH treatments. Data were evaluated for normality (Shapiro-Wilk test) and homogeneity of variances (Levene’s test), and no transformations were necessary. We calculated the percentage change in *NCP* between the initial (pre-manipulation) and final (post-manipulation) values as a function of temperature (ambient vs. warmed), CO_2_ (ambient vs. added), and the interaction between temperature and CO_2_ (temperature × CO_2_). Similarly, we evaluated the change in the internal C:N ratio of the seaweed *Odonthalia* during our experiment as a function of temperature, CO_2_, and temperature × CO_2_. Effects of experimental treatments on community composition were evaluated using PERMANOVA (PRIMER v. 6.1.13 & PERMANOVA + v. 1.0.3, PRIMER-E, Ltd., Ivybridge, UK), and effects on diversity (species richness, Shannon diversity, and evenness as Pielou’s *J*) were examined using general linear models (ANOVA).

## Results

**Fig 3:**
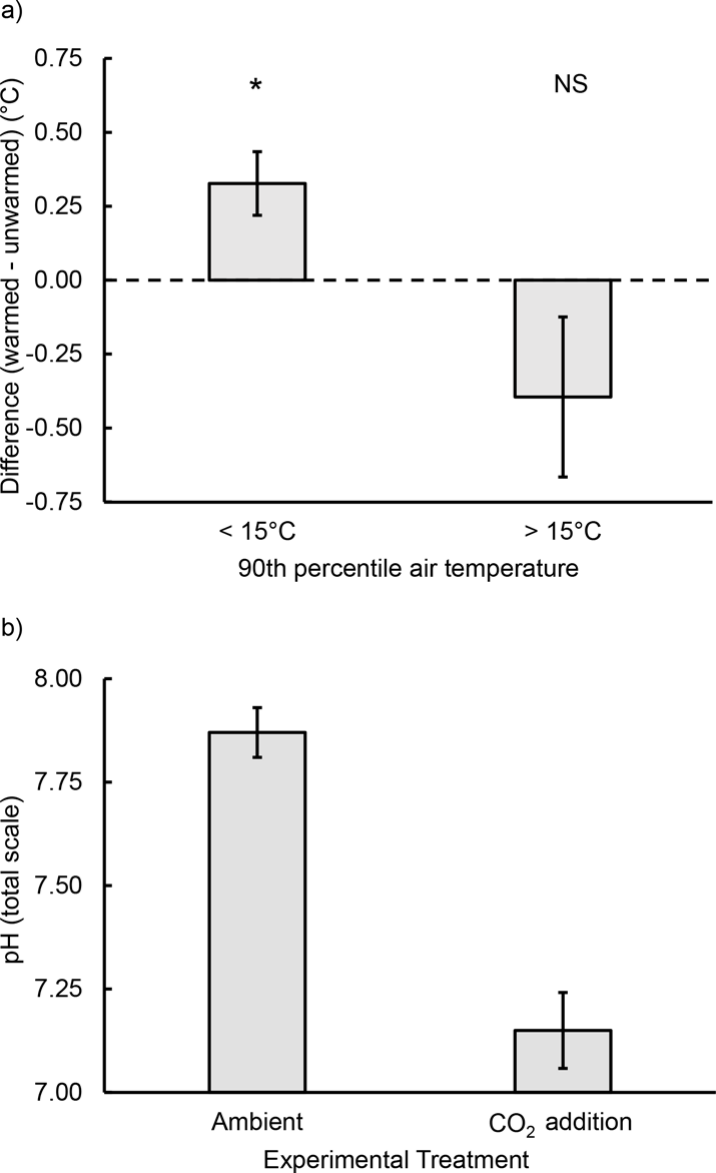
Effects of experimental warming and CO_2_ addition on tide pools. (a) Effects of warming on tide pool temperatures depended on air temperature. Experimental warming increased pool temperatures on cooler days (≤ 15°C) but not when air temperatures were ≥ 15°C. Values are the 90^th^ percentiles of air temperatures (x-axis) and difference between 90^th^ percentile temperatures in warmed *vs*. ambient pools (y-axis) on each day. Statistical significance is indicated: p < 0.05 (*), p > 0.05 (NS). (b) Compared to pools at ambient CO_2_ levels, experimental manipulations led to decreases in average pH (total hydrogen ion scale). Values are means ± SE.

The effects of experimental warming depended on air temperature (F_1,13_ = 8.5, p = 0.012; Fig 3A). On cooler days, when the 90^th^ percentile of air temperatures was ≤ 15°C (the median temperature during our experiment), the 90^th^ percentile of temperatures in warmed pools averaged 0.33 (± 0.11°C higher than in ambient pools (t = 3.1, df = 6, p = 0.023). On warmer days, when temperatures were ≥ 15°C, there was no effect of warming on tide pool temperatures (t = 1.5, df = 7, p = 0.188). Effects of warming on the 90^th^ percentile of water temperatures therefore differed with time (repeated measures ANOVA, within-subject effect: F_14,224_ = 2.6, p = 0.002). However, the effect of temperature did not differ between pools with and without added CO_2_, either overall (between-subjects effect: F_1,16_ = 0.8, p = 0.387) or over time (within-subjects effect: F_14,224_ = 0.1, p = 0.999). Furthermore, daily temperatures did not differ between warmed pools with and without CO_2_ added (p > 0.15 on all days after Tukey adjustment). CO_2_ addition caused pH levels to decrease by approx. 0.7 units in CO_2_ addition pools (7.19 ± 0.09) relative to ambient pools (7.88 ± 0.06) (t = 6.4, df = 18, p < 0.001; Fig 3B). This difference is similar to the change in pH observed in one un-manipulated tide pool during a single daytime low-tide, where pH increased from 7.51 to 8.15 due to passive warming and photosynthetic draw-down of CO_2_. pH levels varied by more than 3 units in un-manipulated pools based on daily measurements during our experiment.

**Fig 4:**
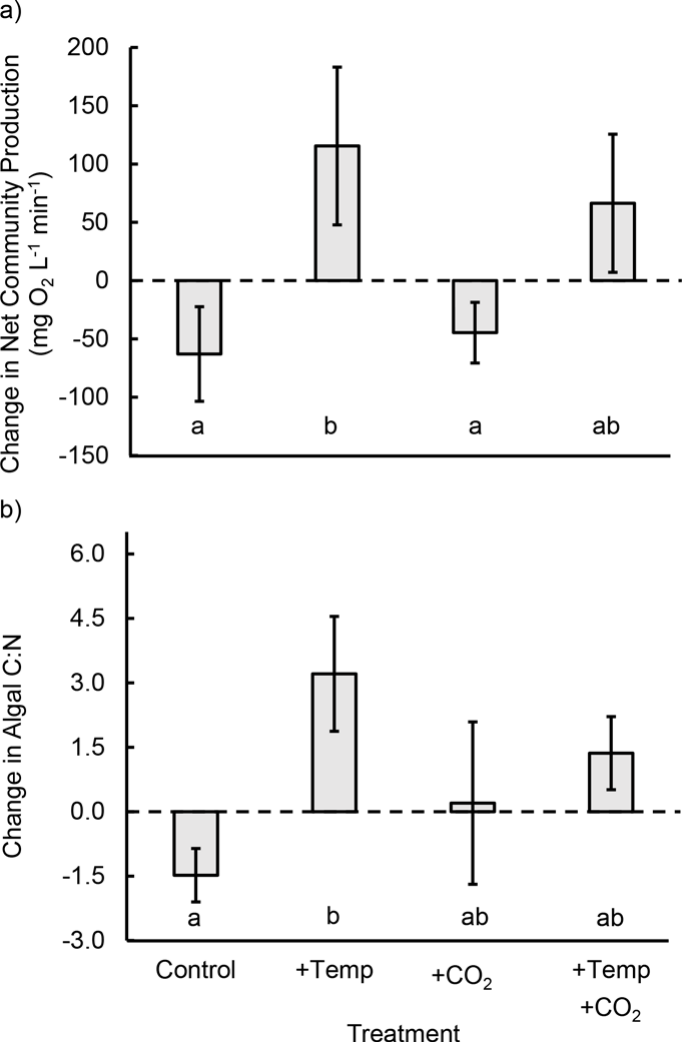
Effects of warming and CO_2_ addition on net community production (*NCP*) and algal C:N. (a) At ambient CO_2_ levels, *NCP* increased in warmed pools. However, when CO_2_ was added, warming did not affect *NCP*. Values are the percentage change between initial (pre-manipulation) and final (post-manipulation) *NCP* measurements. (b) At ambient CO_2_ levels, C:N of the seaweed *Odonthalia* increased in warmed pools. However, when CO_2_ was added, warming did not affect C:N. Values are means ± SE. Letters indicate statistically significant differences after Tukey adjustment for multiple comparisons.

We found a strong effect of experimental warming on *NCP* (F_1,16_ = 8.0, p = 0.012), but no effect of CO_2_ addition (F_1,16_ = 0.1, p = 0.767) or temperature × CO_2_ interaction (F_1,16_ = 0.4, p = 0.518; Fig 4A). Initially, *NCP* did not differ significantly between assigned treatments. After the 16-day manipulation, the percentage change in *NCP* was higher in the warmed treatment than in the control under ambient CO_2_ conditions (p = 0.025 after Tukey adjustment for multiple comparisons), but warming did not affect *NCP* when CO_2_ was added (p = 0.144 after Tukey adjustment). At the end of our 16-d experiment, tide pools where CO_2_ was added had higher O_2_ concentrations than pools with no CO_2_ added (F_1,16_ = 4.73, p = 0.045); however, our *NCP* results did not change when we incorporated O_2_ concentrations as a covariate.

These productivity responses paralleled changes in the physiology of the seaweed *Odonthalia* (Fig 4B). Warming resulted in an increase in the carbon-to-nitrogen ratio (C:N) over the course of our experiment (F_1,16_ = 5.3, p = 0.035) due to a tendency toward both increases in %C and declines in %N in *Odonthalia* collected from warmed pools. There was no effect of CO_2_ on C:N (F_1,16_ < 0.1, p = 0.949), and the effect of temperature on C:N did not change when CO_2_ was added (‘temperature × CO_2_’ interaction; F_1,16_ = 1.9, p = 0.185). The main effect of temperature was driven entirely by a strong effect of warming on C:N under ambient CO_2_ conditions (p = 0.019 after Tukey adjustment) and not by an effect of warming on C:N when CO_2_ was added to pools (p = 0.527 after Tukey adjustment). Thus, as with the *NCP* results (Fig 3A), there was no effect of temperature on C:N when CO_2_ was added. On average, *Odonthalia* cover in tide pools did not change during our experiment, either overall (t = 1.3, df = 19, p = 0.218) or in response to our experimental manipulations (p > 0.30 for all factors [i.e., temperature, CO_2_, temperature × CO_2_, irradiance, and pool volume]). Furthermore, the strong effect of warming on C:N (F_1,17_ = 6.3, p = 0.023) remained after accounting for variation between pools in *Odonthalia* growth (i.e., change in percent cover; F_1,17_ = 4.2, p = 0.056).

**Fig 5:**
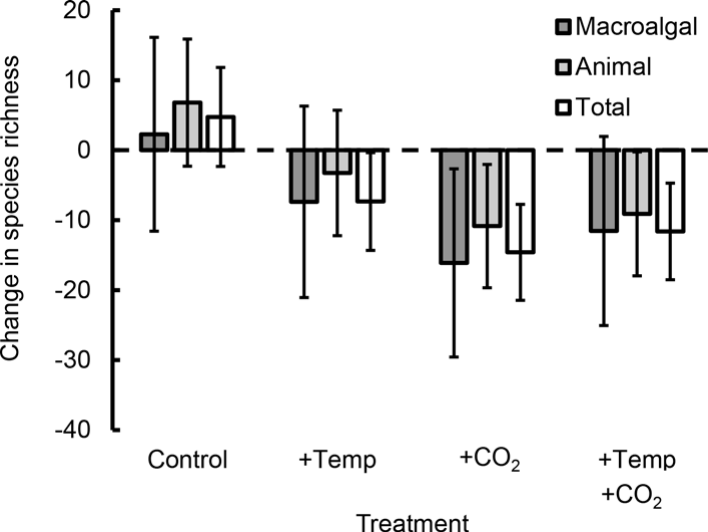
Percentage change in animal, macroalgal, and total species richness in response to experimental treatments. Whereas there was a tendency toward declines in species richness in +CO_2_ treatments, species richness did not respond significantly to our manipulations (p > 0.10 in all cases). Values are least-squares means (± SE) after accounting for tide pool surface area to volume ratio.

There was a tendency toward declines in species richness in +CO_2_ treatments (Fig 5). However, we did not detect any significant responses to our manipulations in any of the community-level metrics, including community composition (PERMANOVA, p > 0.1 for all factors [i.e., temperature, CO_2_, and temperature × CO_2_] and comparisons [i.e., sessile species, mobile species, and algal species]), or richness, evenness, or Shannon diversity (p > 0.1 for all factors and comparisons).

## Discussion

Based on our manipulation of a natural tide pool system, we found that effects of short-term warming and CO_2_ addition occurred in less than a month at the organismal (algal C:N) and ecosystem (*NCP*) levels. These responses were related, with both algal C:N and *NCP* affected by warming but not by addition of CO_2_. A strong response to warming but not to CO_2_ addition is somewhat counterintuitive given that our warming treatments were conservative relative to predicted temperature increases at high latitudes while our CO_2_ additions resulted in a decline in average pH substantially greater than that predicted by the year 2100 [2]. These results also differ from our initial prediction that adding CO_2_ would enhance *NCP*. The organismal and ecosystem-level impacts of warming and CO_2_ addition likely share a common origin in the physiological responses of individual species to the experimental treatments, which translated directly to effects on *NCP*. When there is a direct link between organismal physiology and ecosystem function [18], very rapid responses of the system to climatic changes can occur.

At the organismal level, we found that C:N of the most abundant and widespread seaweed species in our experimental tide pools, the red alga *Odonthalia*, responded to experimental warming. These physiological changes occurred during a low growth period for *Odonthalia*. *Odonthalia* growth typically peaks in the winter and spring, with reproduction occurring in late spring and early summer and individuals then senescing through the summer and fall [19]. Our results were consistent with this seasonality: *Odonthalia* cover did not change during our experiment, suggesting that it was transitioning toward senescence. Furthermore, we found a strong effect of warming on C:N after accounting for change in *Odonthalia* cover, suggesting that this effect was growth-independent.

We can, thus, envision two mechanisms to explain warming-driven increases in algal C:N: loss of proteins and/or increases in carbon accumulation. Warming may have accelerated senescence, which in red algae is associated with a loss of soluble proteins and a corresponding increase in C:N [20]. Higher C:N could also be due to higher rates of carbon accumulation associated with increased *NCP* in warmed pools. Moderate levels of warming often enhance macroalgal photosynthetic rates (e.g., [21,22]), and an increase in *NCP* combined with no growth would lead to accumulation of internal C relative to N. In addition to seasonal changes, *Odonthalia* growth also varies with nutrient availability. Algal C:N averaged 13.6 during our experiment, a value that exceeded the threshold of ~10 for nitrogen limitation in red algae [23] and was much higher than the ratio of ~7.5 measured when *Odonthalia* were actively growing in the spring [24].

The increase in *NCP* in warmed pools was not evident when CO_2_ was added, suggesting that CO_2_ addition depressed the warming-related increase in *NCP*. Potential negative effects of increased CO_2_ concentrations on seaweeds include reduced C affinity, lower HCO_3_^-^ utilization, reduced Rubisco content, and decreased pigment concentrations [25,26], all of which could contribute to decreased *NCP*. Furthermore, these negative effects of CO_2_ addition can interact with temperature. In an indoor mesocosm experiment, Olabarria et al. [27] found that whereas *NCP* was typically higher in warmed mesocosms, the warming effect was more pronounced under ambient CO_2_ conditions and was lower when CO_2_ was elevated. Similarly, C:N was not increased by warming when CO_2_ was added to pools, suggesting that CO_2_ addition limited the effect of warming on C:N. This may reflect a negative effect of CO_2_ addition on *NCP* [25,26], described above, which could reduce rates of carbon accumulation.

Although we detected responses only at the organismal and ecosystem levels, there are several reasons to expect that altering multiple environmental factors could impact community structure (e.g., diversity) and dynamics (e.g., consumption rates) over longer time scales than considered in this study. First, changes in C:N of basal species can affect community dynamics and ecosystem functioning [28]. This represents a mechanism by which organism-level responses to climate change could scale up to successively affect populations, communities, and ecosystems. Second, previous studies suggest that in a longer-term experiment we might expect to see effects of warming and/or CO_2_ addition on rates of herbivory [29,30], predation [31,32], or competition [33,34] or on biodiversity [12,35]. These community-level phenomena have the potential to influence ecosystem-level processes. For example, changes in the diversity of seaweeds on rocky shores can directly affect *NCP* [36]. The importance of these community-mediated effects, relative to the organism-mediated ones we report here, remains unknown, especially in the field.

Given anticipated future changes in both temperature and CO_2_ levels, it is essential to understand not only the independent effects of these factors, but also the potential interactions between them. Factorial manipulations of CO_2_ and temperature in field settings have become an important tool for understanding the effects of climate change on terrestrial communities and ecosystems (e.g., [5]). However, similar factorial manipulations in marine systems have been limited to laboratory and mesocosm studies [27,30,35]. Here, we demonstrate that it is possible to manipulate both temperature and CO_2_ under field conditions and reveal important, interactive effects on both organismal and ecosystem-level processes (e.g., [37]). Further fine-tuning of both CO_2_ delivery and warmers will allow us to match manipulations to regional predictions of future pH and warming. We show that such manipulations are possible, and biological responses are measurable, within the context of a highly dynamic natural system.

## Supporting Information

S1 Tables Datasets supporting this article, including tide pool temperature, pH, community surveys (initial and final), algal C:N, and air temperature. (XLSX)
